# A study on improving nursing clinical competencies in a surgical department: A participatory action research

**DOI:** 10.1002/nop2.485

**Published:** 2020-03-24

**Authors:** Mohammad Afshar, Hamidreza Sadeghi‑Gandomani, Negin Masoudi Alavi

**Affiliations:** ^1^ Trauma Nursing Research Center Kashan University of Medical Sciences Kashan Iran; ^2^ Kashan University of Medical Sciences Kashan Iran

**Keywords:** clinical competence, health services research, nursing

## Abstract

**Aims:**

The purpose of the present study was to improve clinical competencies in nursing staff in a selected surgical department in Kashan/Iran during 2016–2018.

**Design:**

This was a participatory action research.

**Methods:**

This action research was implemented in four steps of problem identification, planning, action and reflection. Interviews, focus groups and observation were used for the qualitative part. Conditions of Work Effectiveness Questionnaire‐II, job satisfaction and patients' satisfaction questionnaires were completed before and after the study. Qualitative content analysis, paired and independent *t* test were used for data analysis.

**Results:**

Three main problems could affect the clinical competencies including professional insufficiency, basic shortages and external influences. Three changes were implemented in the surgical department including improving education, involving nursing students in patients' education and increasing the motivations by peer evaluation and selecting the nurse of the week. The changes significantly increased patients' satisfaction, nurses' job satisfaction and work effectiveness.

**Impact Statement**

- Educational programmes that are presented by the nursing staff are useful in increasing their competencies and satisfaction.
- Pedagogical strategies such as selecting the nurse of the week can increase the motivations and job satisfaction in nurses.
- Nursing students in educational hospitals that have nursing shortage can help nursing staff in patients' education.

## 1 INTRODUCTION

Nurses are the largest group of health professionals, and their competencies have a crucial role in the quality of health services (Fukada, 2018). Competency is a complex and multi‐dimensional concept that has different definitions. Understanding discipline knowledge, mastery of discipline‐specific skills, ability to use sound professional judgement, adherence to professional standards and application of skills and knowledge have been defined as competency in nursing (Church, 2016).

Clinical competency has been a challenging issue in nursing profession (Abbasi, Bahreini, Yazdankhah Fard, & Mirzaei, 2017). The feeling of low competency could decrease job satisfaction and increase occupational withdrawal; it also influences the quality of care (Wade et al., 2008). On the contrary, feeling competent could decrease burnout and increase self‐confidence (Greco, Laschinger, & Wong, 2006).

Nursing care in surgical departments needs considerable clinical competencies. Surgery is a traumatic procedure, and patients need special care. (Fero, Witsberger, Wesmiller, Zullo, & Hoffman, 2009; Majid et al., 2011). Many patients confront complex situations, and nurses should be competent in making urgent and correct clinical decisions in surgical wards (Jangland, Nyberg, & Yngman‐Uhlin, 2017). Besides, infection is an important issue in surgical departments and surgical nurses must be competent in implementing infection control strategies (Troughton et al., 2019).

## 2 BACKGROUND

Studies in Iran show that nurses estimate their clinical competency to be at a moderate or good level (Adib Hajbaghery & Eshraghi Arani, 2018; Elhami, Ban, Mousaviasl, & Zahedi, 2018; Karami, Farokhzadian, & Foroughameri, 2017). In a study conducted in Iran, job satisfaction and clinical competency were good and these two variables had a statistically significant and direct relation (Abbasi et al., 2017). However, in prevention of hospital infections, their competencies were at a novice level (Teymourzadeh, Bahadori, Fattahi, Khodadost, & Shokri, 2019). Another study showed that most of the nurses had undesirable competency in spiritual care (Adib‐Hajbaghery, Zehtabchi, & Fini, 2017). These studies show that there are controversies in clinical competencies of the nurses in Iran.

There are few studies about the improvement of professional competencies in nursing. A study in Korea showed that critical reflection programme can improve critical thinking and communication abilities among novice nurses (Kim, Min, Kim, & Shin, 2018). Empowering nurses and supervisors with workshop and educational materials could improve the overall patient safety culture in Iran (Amiri, Khademian, & Nikandish, 2018). Another study showed an externship programme and a corporate‐academic cooperation programme can enhance junior college students' nursing competence and retention rates (Tseng, Hsieh, Chen, & Lou, 2013). Most of these researches are quasi‐experimental studies that use external interventions to create temporary outcomes. Mixed methods within a Participatory Action Research (PAR) provide a greater understanding of the situation and ensure realistic interventions to influence behavioural change in specific settings (Sendall, McCosker, Brodie, Hill, & Crane, 2018).

Participatory action research is a systematic approach that assists participants in articulating their research needs and developing strategies to address them. Participants are equal partners and often named as co‐researcher. Empowering and change in practice are the aims of PAR (Cusack, Cohen, Mignone, Chartier, & Lutfiyya, 2018). This research method is proper for making changes and quality improvement. This PAR was designed to improve the clinical competency of nurses and its application in nurses working in a selected surgical department.

### 2.1 Design of the study

**FIGURE 1:**
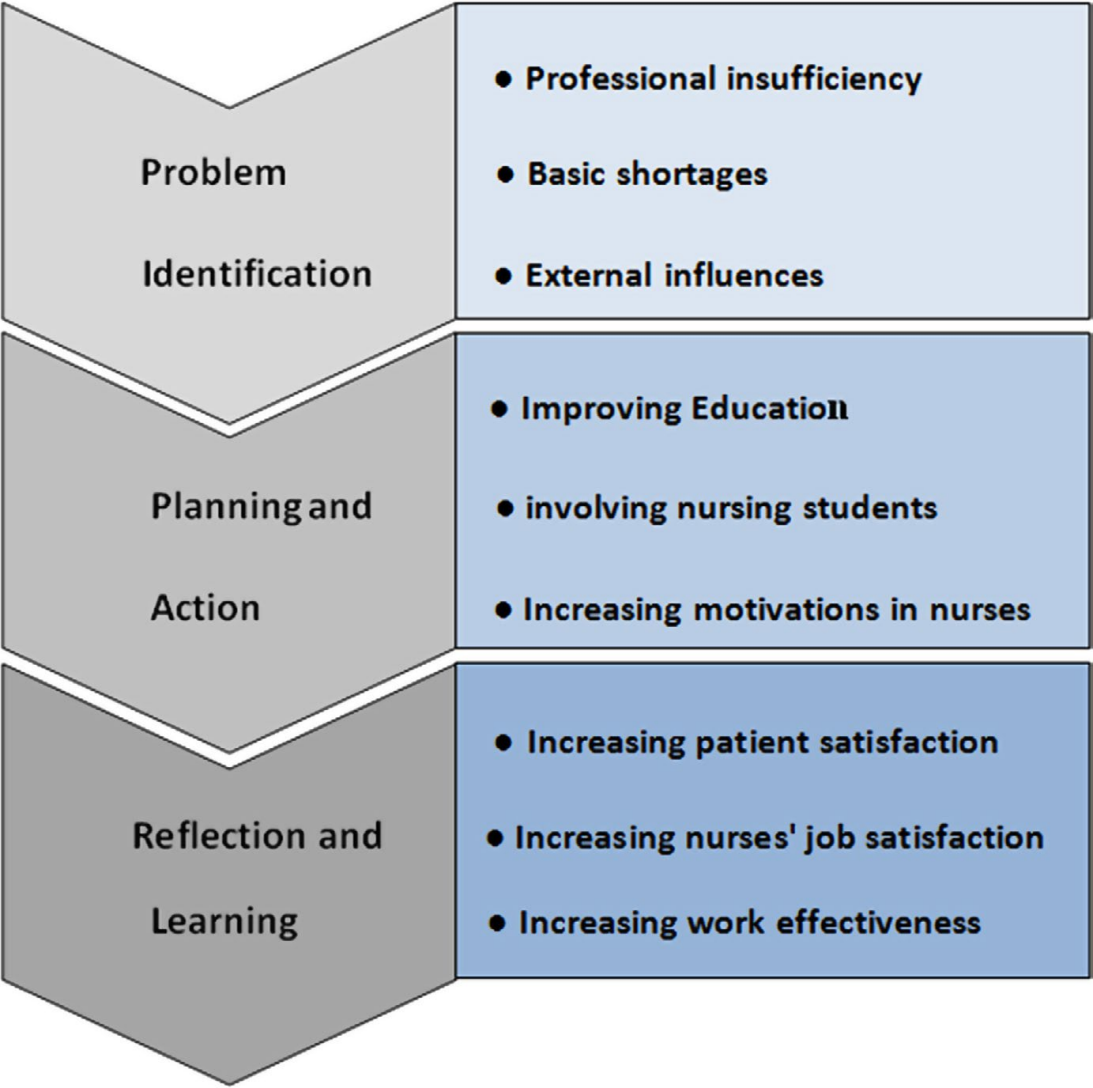
The steps of participatory action research

The design of the present study was PAR. The idea of the study was derived from the nursing practice and observing that nurses were not using their clinical competencies in their usual work. Four phases of problem identification, planning, action and reflecting were used in this study (Figure 1) (Nhamo, 2012). This manuscript has been prepared according to the Standards for Quality Improvement Reporting Excellence (SQUIRE 2.0)—See Appendix S1.

## 3 METHODS

### 3.1 Study settings and participants

The setting of the PAR was a surgical department in a general hospital in Kashan/Iran. This ward provides services to male patients with orthopaedic and urology surgeries. In average, length of stay in this ward is 3 days. The first cycle of this PAR was started in September 2016 and ended in May 2018.

A head nurse and all the staff nurses, a matron, supervisors and two nursing faculty members and a PhD student of Kashan University of Medical Sciences participated in this study. The head of the department was a surgeon invited in the first meeting to get familiar to the project and to facilitate the communication between PAR and surgery team. An orthopaedic surgeon also took part in a meeting related to the interventions and provided his suggestions.

### 3.2 Data collection

The qualitative and quantitative data were collected and analysed simultaneously.

*Qualitative data:* Interviews, focus groups and observation were used for data collection. The head nurse and staff nurses were interviewed in the beginning and in the end of the study. The interviews were semi‐structured and were held in a room in the surgical department. In the beginning of the study, the interview started with statements such as: Please explain one day of your work; how do you feel about your clinical competencies? What are your experiences in providing good quality nursing care? How do you think you can reach to the maximum of your clinical competencies? In the end of the study, the interviews were focused on the participants' experiences of PAR and its outcomes. The patients were also interviewed about their experiences in receiving nursing care and how they feel about the competencies of the nurses.

There were 16 focus groups and five meetings during the study period. The first focus groups were about the analysis of the interviews and problem identification. Later, focus groups were held to decide about the possible interventions, to discuss the effects of the interventions and to solve the practical problems. The head nurse scheduled focus groups in a way that all the nurses from all the shifts could attend at least one of the meetings. All the interviews and meetings were audio‐taped and transcribed.

The coordinator of the study (PhD student) was present in the surgical department at different shifts. He observed the behaviours of the nurses when they were providing care to the patients. The field notes were about the clinical behaviours of the nurses in the surgical ward and their use of competencies.

*Quantitative date:* Three questionnaires were completed at the beginning of the study in September 2016 and at the end of the study in May 2018. Two questionnaires were completed by all the staff nurses to measure their work effectiveness and job satisfaction. The third questionnaire was completed by the patients to measure their satisfaction with nursing care. It was assumed that if the nurses' competencies would improve during PAR, they will provide better care, and this will be reflected on patients' satisfaction through its increase.

- The Conditions of Work Effectiveness Questionnaire‐II (CWEQ‐II): This is a 19‐item questionnaire that measures respondent's perceived access to opportunity, information, support and resources and perceived formal and informal power in their work environment. This questionnaire has been developed by Laschinger, Finegan, Shamian, and Wilk (2001). The items have a 5‐point Likert scale, with higher scores representing higher levels of empowerment; possible scores range from 6–30. The reliability coefficient of this questionnaire was reported to be 0.89 (Armstrong, Laschinger, & Wong, 2009). This questionnaire has been translated to Farsi. Its content validity index (CVI) and Cronbach's *α* coefficient have been reported to be .94 and .84, respectively (Sadeghi‐Gandomani, Alavi, & Afshar, 2019). Cronbach's *α* coefficient of this questionnaire in current study was .78.
- The Job Satisfaction Survey (JSS): This questionnaire, developed by Spector in 1985, has 36 items that is ranked on a 6‐point Likert scale ranging from “strongly disagree” to “strongly agree.” The subscales are as follows: pay, promotion, supervision, fringe benefits, contingent rewards, operating procedures, co‐workers, nature of work and communication. Scores on the total job satisfaction can range from 36–216. Cronbach's *α* coefficient of this questionnaire has been reported to be .8 (Spector, 1985). The Farsi translation of this questionnaire has been validated by Gholami et al. This questionnaire has good construct validity, and its Cronbach's *α* has been reported to be .865 (Gholami, Talebiyan, Aghamiri, & Mohammadian, 2012). Its Cronbach's *α* coefficient in this study was .82.
- The patient satisfaction questionnaire: This questionnaire has been developed by Ghods et al in Farsi language in 2011. It has 45 items and evaluates patient's satisfaction in four dimensions of empathy in caring, proficiency, negligence and cleanliness. The items have a five‐point Likert scale from completely disagree to completely agree. The possible range of scores is 45–225, with higher scores indicating higher patient satisfaction. The questionnaire has a good construct validity, and its internal consistency has been reported to be 0.7 (Ghods, Mohammadi, Vanaki, & Kazemnejad, 2011). Its Cronbach's *α* coefficient in this study was .74. Based on the ability to detect 15 differences in the score of patients' satisfaction (effect size), *SD* of 48.01 (Sadeghi Gandomani, Masoudi Alavi, & Afshar, 2018), power of .80 and *α* of .05, the minimum sample size was determined to be 205. A total of 250 patients completed the questionnaire before and after the study. The patients who were hospitalized for at least 48 hr and agreed to complete the questionnaires were enrolled in the study sequentially.

### 3.3 Data analysis

The qualitative content analysis suggested by Graneheim and Lundman (2004) was used for qualitative data analysis. The MAXQD software version 10 was used to manage the data. The content of the interviews was completely transcribed. To get a general idea, the transcripts were read by all three authors several times. Then, the text about the participants' experiences of competencies, problem identification and suggested interventions were extracted, and sub‐categories and categories were discussed by all three authors until consensus was reached.

The variables about the nurses' work effectiveness and satisfaction and patients' satisfaction were presented descriptively by frequency, mean and *SD* and the differences of the variables from the beginning and the end of the study were analysed using statistical tests including paired *t* test and independent *t* test. The data were analysed using SPSS version 16. A *p*‐value < .05 was accepted as statistically significant.

## 4 RESULTS

There were 14 nurses in the ward: nine were female and five were male. All of them had bachelor's degree in nursing. The average age and work experience of the participants were 36.7 (*SD* 6) and 12.7 (*SD* 3) years, respectively.

### 4.1 Problem identification

*The qualitative data:* There were 20 interviews with staff nurses, head nurse, supervisors, matron and surgeons. Four patients were also interviewed. The interviews were between 32–85 min with mean duration of 56 ± 12 min. Four focus groups were held. Six field notes were also recorded. After analysing the qualitative data, three main problems were identified (Table 1):

- Professional insufficiency: Lack of professionalism in nurses, ineffective management and insufficient education were the sub‐categories of this problem. A nurse said: “Our priority must be education. When I don't know the things, how am I supposed to give care to the patients or feel competent? The classes and workshops that are held for us don't have good quality. Most of the time nurses just fill the presence list and leave the class. It is just like a routine work to say we had 10 workshops, and nobody cares about the quality of the programs.”
- Basic shortages: Shortage in the nursing staff, supplies and substandard facilities were some of the basic problems. A nurse said: “We should change the IV sets every day, but we change it every 4 days because there is a shortage of the equipment. We don't have a standard dressing set, nothing is standard, how can I feel competent when I know that I am not doing my job well because I don't have the standard equipment. Mostly, I don't have enough time because this is a surgical ward all of our patients need special attention, but we are only 14 nurses in this ward. A ward like this should have at least 23 nurses.”
  - A patient said: “The nurses change regularly I don't know their names. They don't introduce themselves. They are always in hurry and don't talk to me. They injected antibiotic to my vein so fast that I felt vertigo; I know it should be injected slowly. They do things fast and leave like they are in a race.”
- External influences: Physician‐centred services, low education of the patients and wrong policies in the ministry of health were the problems that were mentioned by the nurses. A nurse said: “In the ministry of health they just care about the benefits of the doctors, they don't pay attention to the nurses. The income of the nurses and the surgeons has no such difference in any other parts of the world. The difference is unbelievable. All the nurses had lost their motivations and interests. Why should I kill myself for patients when surgeons take all the benefits?”

**TABLE 1:** Categories and sub‐categories derived from qualitative data analysis

Sub‐categories	Categories
Lack of professionalism in nurses	Professional insufficiency
Ineffective management	
Insufficient education	
The shortage in nursing staff	Basic shortages
The shortage in supplies	
Substandard facilities	
The physician‐centred services	External influences
Low education of the patients	
Wrong policies in the ministry of health	

*The quantitative data:* All the nurses completed the Conditions of Work Effectiveness Questionnaire‐II and job satisfaction questionnaires. The score of work effectiveness was 16.25 ± 3.22 that shows a medium effectiveness. The score of job satisfaction was 111.5 ± 22.78 that showed low satisfaction. The mean score of the patient satisfaction was 136.05 ± 48.1 that showed moderate satisfaction (Table 2).

**TABLE 2:** Patients satisfaction, nurses' job satisfaction and work effectiveness before and after first cycle of the participatory action research

Variables	Before the interventions	After the interventions	*t*	*p*
The conditions of work effectiveness	16.25 ± 3.22	25.31 ± 4.13	35.89	.0001
Nurses' job satisfaction	111.5 ± 22.78	163.34 ± 26.41	51.49	.0001
Patient satisfaction	136.05 ± 4.6	159.4 ± 18.81	19.03	.0001

### 4.2 Planning and action

In four focus groups, the problems and possible changes were discussed with nurses. The suggestions were analysed, and the feasibility of the plans was reviewed. Three change plans that were more applicable were selected by consensus in action research team. The action plan was designed for each change, and it was performed in 7 months (Table 3). The progress and possible modifications were evaluated in three focus groups:

- Educational changes: All the nurses accepted that there was a need to increase the clinical competencies by education. The nurses chose self‐learning, journal club and in‐ward short educational rounds as the methods for education. The educational subjects that were suggested by the nurses were divided between the AR team. All the members including the staff nurses presented the educational sessions inside the ward with the time frame that had been planned by the head nurse. The timetable of the educational programmes including journal clubs and educational rounds could be seen in the nursing station. Programmes were registered to the continuous medical education programme (CME) so that nurses could benefit from the hours. The mediator of the AR designed a telegram channel that all the educational programmes and films could be shared between the members. The ward did not have a library, so a small library with current references in nursing was prepared for the nurses.
- Involving the nursing students: The AR team noticed that the interaction between the nurses and the patients was inadequate, and patients were not receiving the necessary information. This was largely due to nursing shortage. The team decided to involve nursing students in patient education. With the support of the nursing faculty, the nursing students were obliged to have an active role in patients' education from admission to discharge. The head nurse and the mediator prepared the students and held several educational classes for the students.
- Increasing motivations of the nurses: The research team noticed that nurses were frustrated and thought that their services were not respected by others. The research team started a programme to increase their motivations. A peer group evaluation was designed in the ward. The nurses completed a small questionnaire with five items. They were asked to write the name of one of their colleagues that was doing the best nursing care, was the most cooperative, was the most disciplined, had more empathy with the patients and was the most knowledgeable and skilful during the past week. Every week, a nurse was selected according to the peer evaluation and small present along with acknowledgement were given to the nurse by the head nurse.

**TABLE 3:** Action plan to improve clinical competencies in the nursing staff

Objectives	Action plan
Improving education	Involvement of the nurses in selecting educational subjects
	Education through self‐learning
	Education trough journal clubs
	In‐ward short educational rounds
	Teaching by peer group
	Educational sessions in all shifts
	Registration of educational sessions in the continuous medical education
	Providing a library in the department
	Educational telegram canal
Involving the nursing students	Coordination with the nursing faculty
	Preparing the content of patients' education
	Preparing the nursing students for patients' education
Increasing the motivations of nurses	Designing a scale for peer group evaluation
	Using peer group evaluation
	Selecting the nurse of the week according to peer group evaluation
	Encouraging the selected nurses

### 4.3 Reflection

During action research and in the end of the programme, the reflections were gathered through focus groups and interviews. Some changes were made in the planning according to the reflections. Some educational subjects were added to the programmes such as communicational skills and new products in wound care. Overall, most of the reflections were positive and nurses stated that the changes had improved their feeling of clinical competency. A nurse said: “Educations were very helpful. The best thing was that we should not leave the ward and our colleagues knew our educational needs better. For example, the neurovascular assessment and capillary refill are very important in orthopaedic patients. We didn't use to pay attention to that but now we assess the patients. Now we know more about the wound care and our self‐confidence has improved.”

Another nurse said: “Our communication with the head nurse and supervisor has improved. We think we are more respected by the patients and the surgeons. Our teamwork is better now. I have been selected as the nurse of the week two times by my colleagues (with laugh), I think they know I am a good nurse.”

In the end of the AR, all the nurses completed the questionnaires once again. A total of 250 patients also completed the patient satisfaction questionnaire. The score of work effectiveness was increased to 25.31 (*SD* 4.13) from 16.25 (*SD* 3.22) that showed a statistically significant improvement (*p* = .0001). The score of job satisfaction was also increased to 163.34 (*SD* 26.41) that showed a statistically significant difference (*p* = .0001). The patient satisfaction was 136.05 (*SD* 48.1) that was increased to 159.4 (*SD* 18.81) (*p* = .0001) (Table 2).

## 5 DISCUSSION

Nurses must provide comprehensive care to address complex and diverse needs of the patients. It is important for nurses to improve their competency and use it in their daily practice (Fukada, 2018). This PAR showed that simple interventions that involved nurses could improve clinical competencies. Action research is a practical way for implementing changes and reforms. This method has been used successfully in different settings. In Vietnam, AR has been used for educational reform and changing the nursing education into competency‐based nursing curriculum (Chapman, Lewis, Osborne, & Gray, 2011). Another PAR in Iran was relatively successful in creating a collaborative partnership between educators and practitioners to identify and enhance educational practices for the first clinical experience of the nursing students (Asadizaker, Abedsaeedi, Abedi, & Saki, 2016). Most of the action researches in the nursing have been focused on nursing education. Nurses in hospital departments have many problems and confront many challenges that PAR might be helpful in finding practical solutions for them. Using PAR in hospital departments could be helpful in improving the quality of care and work conditions.

This action research tried to bring the supervisors, head nurse, staff nurses and faculty members closer to each other, to achieve a common sense and consensus on how the clinical competencies can improve. Peer networks are one of the informal means for access to information support and resources. Sharing power, enhancing communication and participatory decision‐making are the prerequisite of successful change and this can improve competencies (Macphee, Skelton‐Green, Bouthillette, & Suryaprakash, 2012). This dynamic collaboration between nursing staff and faculty members can improve professional dignity and decrease the gap between theory and practice in nursing.

Delivery of safe and high‐quality care demands reliable teamwork and collaboration of the clinicians and the administrative staff (Rosen et al., 2018). In this study, the patients were also more satisfied with the nursing care after the action research. A study showed that verbal communication of the nurses with the patients had a statistically significant correlation with patient satisfaction (Sadeghi Gandomani et al., 2018). Involving nursing students that helped the staff nurses in patients' education and workshops on effective communication might have influenced the patient satisfaction in this PAR. Improving the quality of care that has been reflected in increasing patients' satisfaction is one of the desired outcomes in nursing profession in this study.

Improving work environment that was perceived by the nurses can result in greater professionalism, improved job satisfaction and job engagement (Fan, Zheng, Liu, & Li, 2016). Work environments that support professional nursing practice result in more positive outcomes for both the nurses and the patients (Spence Laschinger, Wilk, Cho, & Greco, 2009). PAR, regardless of its objectives and results, could improve the work environment. This could be an outcome that needs further attention and investigation.

A qualitative research showed that being valued as a learner, a team member and a person was a prominent factor in feeling empowered and competent (Bradbury‐Jones, Sambrook, & Irvine, 2011). In this study, interventions such as selecting the nurse of the week by co‐workers and active participation in educational programmes might make nurses to feel more valued and increase their professional dignity; this might explain the increase in job satisfaction.

Job satisfaction is an important variable and predictor of wellness in the work environment. It has a profound impact on the productivity and the effectiveness of the services (Tsounis & Sarafis, 2018). A study showed that empowerment, physician–nurse relationship and organizational support/trust were the most important predictors of job satisfaction (Kretzschmer et al., 2017). Current PAR successfully increased job satisfaction in the nurses. This might be due to the increase in the sense of effectiveness.

Nurses need to be informed about the current best practices, knowledge and skills to be able to provide safe and effective patient care. Learning opportunities that use online environment are common in continuing the education for nurses and other health professionals (Green & Huntington, 2017). The educational interventions were the cornerstone of this PAR. The telegram network also helped us to send educational films and texts to the nurses.

Nurses mentioned some basic shortages such as nursing shortage and substandard devices as obstacles in clinical competencies. Nursing shortages have been a serious problem in health services. In the United States, it has been predicted that 260,000 positions for RNs will remain unfilled by the year 2025 (Kretzschmer et al., 2017). Nursing shortages cause heavier workloads, poor working conditions and increased stress on nurses. Overcoming these basic shortages was beyond the abilities of the research team. But with collaboration of the nursing faculty, nursing students were involved in patient care especially in patient education.

## 6 CONCLUSION

The target of the present study was to improve clinical competencies and researchers decided to measure the outcomes of this improvement including job satisfaction, patients' satisfaction and nurses' work effectiveness. This PAR successfully improved the outcome variables. There were some other outcomes that could be further studied such as inter and intra‐professional relationships and nurses' burnout that might be considered in future studies. The patients who completed the satisfaction questionnaire were different in the beginning and end of the study, so the change that has been noticed must be interpreted cautiously. The research environment was a single surgical department with limited nursing staff; the interventions were planned according to their feasibility and applicability. We recommend more PAR with same objectives in other hospital departments.

## 7 RELEVANCE TO CLINICAL PRACTICE

This PAR had statistically significant outcomes such as increasing patients' satisfaction, nurses' job satisfaction and work effectiveness. The simple steps that involve nurses can have profound outcomes in clinical settings and improve the quality of care and professional dignity. The cooperation between the faculty of nursing and the hospital departments can have a crucial role in solving the nursing problems in hospital departments and decrease the gap between research and practice. In our experience, the PAR can be used successfully in clinical settings.

## CONFLICT OF INTEREST

There is no conflict of interest in this study.

## AUTHOR CONTRIBUTIONS

All the authors meet all of the authorship criteria for this paper. We three conceived and designed the study, implemented and led data collection, conducted all data analyses, interpreted results and wrote the entirety of the manuscript. We are all appropriately listed on the by‐line as authors, and we have agreed to the contents of the manuscript.

## ETHICAL APPROVAL

This study has been approved by the research ethics committee of Kashan University of Medical Sciences with the ethical code: IR.KAUMS.MEDNT.REC.1396.92. All the necessary permissions were obtained from the Kashan University of Medical Sciences and Shahid Beheshti hospital before starting the study. Written informed consent was obtained from all the patients who completed the questionnaires and nurses who participated in the PAR team, including consent form for audio taping and transcribing the interviews and focus groups. Helsinki declaration in ethical codes was respected in all the stages of the study.

## Supporting information

Appendix S1
