# Supplementary material for: A study on improving nursing clinical competencies in a surgical department: A participatory action research
Source: Nurs Open. 2020 Mar 24;7(4):1052–9. doi: 10.1002/nop2.485 (PMC7308675; doi:10.1002/nop2.485)
Supplement: Supplementary file 1 — Appendix S1 [file NOP2-7-1052-s001.doc]

**Appendix S1**

| **Revised Standards for Quality Improvement Reporting Excellence (SQUIRE 2.0)**  **September 15, 2015** | | |
| --- | --- | --- |
| **Text Section and Item Name** | **Section or Item Description** | |
| **Notes to authors** |  The SQUIRE guidelines provide a framework for reporting new knowledge about how to improve healthcare   The SQUIRE guidelines are intended for reports that describe system level work to improve the quality, safety, and value of healthcare, and used methods to establish that observed outcomes were due to the intervention(s).   A range of approaches exists for improving healthcare. SQUIRE may be adapted for reporting any of these.   Authors should consider every SQUIRE item, but it may be inappropriate or unnecessary to include every SQUIRE element in a particular manuscript.   The SQUIRE Glossary contains definitions of many of the key words in SQUIRE.   The Explanation and Elaboration document provides specific examples of well-written SQUIRE items, and an in-depth explanation of each item.   Please cite SQUIRE when it is used to write a manuscript. | |
| **Title and Abstract** | | |
| **1. Title** | | Indicate that the manuscript concerns an initiative to improve healthcare (broadly defined to include the quality, safety, effectiveness, patient-centeredness, timeliness, cost, efficiency, and equity of healthcare)  Page 1: **Improving Nursing Clinical Competencies in a Surgical Department: A Participatory Action Research Study** |
| **2. Abstract** | | a. Provide adequate information to aid in searching and indexing  b. Summarize all key information from various sections of the text using the abstract format of the intended publication or a structured summary such as: background, local problem, methods, interventions, results, conclusions  Page 1: |
| **Introduction** | | *Why did you start?* |
| **3. Problem Description** | | Nature and significance of the local problem  Page 3: The experiences of the researchers show that Iranian nurses suffer from low clinical competencies especially in surgical wards that need high level of clinical skills. Besides nurses must be confident on their competencies and should be supported to use their capabilities in nursing care. |
| **4. Available knowledge** | | Summary of what is currently known about the problem, including relevant previous studies  Page 2: There are a few studies about the improvement of professional competencies in nursing. Most of them are quasi experimental studies that use external interventions to create temporary outcomes |
| **5. Rationale** | | Informal or formal frameworks, models, concepts, and/or theories used to explain the problem, any reasons or assumptions that were used to develop the intervention(s), and reasons why the intervention(s) was expected to work  Page 3: Participatory action research (PAR) is a research style that is grounded in trust, mutuality and openness. The starting point of the PAR is an inquiry into a concern that forms the research question. Empowering, and change in practice are the aims of PAR. It is possible through collaboration between practitioners and researchers. |
| **6. Specific aims** | | Purpose of the project and of this report  Page 3: This PAR was designed to improve clinical competencies and its usage in nurses working in a selected surgical department. |
| **Methods** | | *What did you do?* |
| **7. Context** | | Contextual elements considered important at the outset of introducing the intervention(s)  Page 3: The setting of the PAR was a surgical department in Shahid Beheshti hospital in Kashan/Iran. Kashan is a city with about 400000 residents that is located in the edge of central desert of Iran. Shahid Beheshti Hospital is the only educational general hospital of the city with 400 beds. |
| **8. Intervention(s)** | | a. Description of the intervention(s) in sufficient detail that others could reproduce it  b. Specifics of the team involved in the work  Pages 9-11: Educational changes, involving nursing students, increasing motivations. |
| **9. Study of the Intervention(s)** | | a. Approach chosen for assessing the impact of the intervention(s)  b. Approach used to establish whether the observed outcomes were due to the intervention(s)  Pages 4-6: Using both qualitative and quantitative methods. |
| **10. Measures** | | a. Measures chosen for studying processes and outcomes of the intervention(s), including rationale for choosing them, their operational definitions, and their validity and reliability  b. Description of the approach to the ongoing assessment of contextual elements that contributed to the success, failure, efficiency, and cost  c. Methods employed for assessing completeness and accuracy of data  Pages 5-6: Using three questionnaires of job satisfaction, patients satisfaction and work effectiveness. |
| **11. Analysis** | | a. Qualitative and quantitative methods used to draw inferences from the data  b. Methods for understanding variation within the data, including the effects of time as a variable  Pages 6-7: Qualitative content analysis and The variables about the nurses' work effectiveness and satisfaction and patients' satisfaction were presented descriptively by frequencies and mean and standard deviation and the differences of the variables from the beginning and the end of the study were analyzed using statistical tests including pair t test and independent t test. |
| **12. Ethical Considerations** | | Ethical aspects of implementing and studying the intervention(s) and how they were addressed, including, but not limited to, formal ethics review and potential conflict(s) of interest  Page 7: The research project approved by the Research ethics committees at the Kashan University of Medical Sciences with the ethical code: IR.KAUMS.MEDNT.REC.1396.92. All the necessary permissions were obtained from university and the Shahid Beheshti hospital before starting the study. The participants and patients were informed about the study objectives. Helsinki declaration in ethical codes was respected in all the stages of the study. |
| **Results** | | *What did you find?* |
| **13. Results** | | a. Initial steps of the intervention(s) and their evolution over time (*e.g.*, time-line diagram, flow chart, or table), including modifications made to the intervention during the project  b. Details of the process measures and outcome  c. Contextual elements that interacted with the intervention(s)  d. Observed associations between outcomes, interventions, and relevant contextual elements  e. Unintended consequences such as unexpected benefits, problems, failures, or costs associated with the intervention(s).  f. Details about missing data  Pages 7-12 |
| **Discussion** | | *What does it mean?* |
| **14. Summary** | | a. Key findings, including relevance to the rationale and specific aims  b. Particular strengths of the project  Page 12: This PAR showed that simple interventions that involve nurses can improve clinical competencies. |
| **15. Interpretation** | | a. Nature of the association between the intervention(s) and the outcomes  b. Comparison of results with findings from other publications  c. Impact of the project on people and systems  d. Reasons for any differences between observed and anticipated outcomes, including the influence of context  e. Costs and strategic trade-offs, including opportunity costs  Pages 12-14 |
| **16. Limitations** | | a. Limits to the generalizability of the work  b. Factors that might have limited internal validity such as confounding, bias, or imprecision in the design, methods, measurement, or analysis  c. Efforts made to minimize and adjust for limitations  Pages 14-15: The patients who completed the satisfaction questionnaire were different in the beginning and end of the study, so the change that has been noticed must be interpreted cautiously. The research environment was a single surgical department with limited nursing staff; the interventions were planned according to their feasibility and applicability. |
| **17. Conclusions** | | a. Usefulness of the work  b. Sustainability  c. Potential for spread to other contexts  d. Implications for practice and for further study in the field  e. Suggested next steps  Pages 14-15: The target of the study was to improve clinical competencies, and researchers decided to measure the outcomes of this improvement including job satisfaction, patients' satisfaction and nurses' work effectiveness. This PAR successfully improved outcome variables. |
| **Other information** | |  |
| **18. Funding** | | Sources of funding that supported this work. Role, if any, of the funding organization in the design, implementation, interpretation, and reporting  Page 2 of title page: This study has been funded by deputy of research in Kashan University of Medical Sciences as a PhD thesis in Nursing with the grant number Reg. Code: 96187. |
